# Immediate Effects of Aerobic Exercise and Whole-Body Vibration on Fat Oxidation, Lipid Mobilization, and Cardiovascular Response in Individuals with Obesity

**DOI:** 10.3390/jcm13010044

**Published:** 2023-12-21

**Authors:** Sothida Nantakool, Khanittha Punturee, Supatcha Konghakote, Cattaleeya Sitthichoke, Kochaphan Phirom, Busaba Chuatrakoon

**Affiliations:** 1 Environmental-Occupational Health Sciences and Non Communicable Diseases Research Center, Research Institute for Health Sciences, Chiang Mai University, Chiang Mai 50200, Thailand; sothida.n@cmu.ac.th (S.N.); kochaphan.ph@cmu.ac.th (K.P.); 2 Center Research Unit of Associated Medical Sciences (AMS-CRU), Faculty of Associated Medical Sciences, Chiang Mai University, Chiang Mai 50200, Thailand; khanittha.taneyhill@cmu.ac.th; 3 Department of Physical Therapy, Faculty of Associated Medical Sciences, Chiang Mai University, Chiang Mai 50200, Thailand; supatcha.k@cmu.ac.th (S.K.); cattaleeya.sit@cmu.ac.th (C.S.)

**Keywords:** obesity, whole-body vibration, aerobic exercise, fat oxidation, lipid mobilization, cardiovascular response

## Abstract

Among obesity, cumulative fat and poor physical activity are risk factors for cardiovascular disease. Due to the limit in performing aerobic exercise (AER), whole-body vibration (WBV) as a passive form of exercise is an alternative therapeutic strategy. Herein, this study aimed to compare the immediate effects of AER and WBV on metabolic and cardiovascular responses, and dyspnea level in obesity. Forty-nine eligible obesities performed both AER and WBV, with a random order assignment (age = 28.94 ± 11.39 years). Fat oxidation, cardiovascular parameters (i.e., heart rate (HR) and blood pressure (BP)), and dyspnea level (i.e., rating perceived exertion (RPE)) were measured during exercise, while lipid mobilization (i.e., triglycerides) was collected pre- and post-exercise. Fat oxidation rate in AER was significantly higher than in WBV. Significantly increased fat oxidation rates were shown in both groups (within-group analyses) (also shown in females aged 20–45). Triglyceride levels between AER and WBV were similar. A significant decrease in triglyceride levels was only observed in WBV (within-group change). HR and RPE in AER were significantly higher than in WBV (*p* < 0.05). HR and RPE were significantly increased throughout both AER and WBV, while systolic blood pressure was only significantly elevated in AER (whining-group analyses). WBV may facilitate fat oxidation (particularly in females aged below 45), induce lipid mobilization, and reduce interference on cardiovascular parameters in obesity.

## 1. Introduction

Obesity is a concerned health problem worldwide [1]. Evidence has reported a three-fold increase in the incidence of obesity compared to 40 years ago across countries [2]. In Thailand, a 10% increase in obesity has been documented in the population within 5 years (2012–2018) [3]. Obesity is characterized by excessive fat accumulation, which contributes to health risk [2]. Excessive fat is a factor for cardiometabolic risks [4,5]. Hypertriglyceridemia in obesity plays an initial role for other lipid abnormalities [4] and further promotes atherosclerosis [6]. In addition, obese adipose tissue links to type 2 diabetes via the development of insulin resistance [7,8]. One of the modifiable factors that plays an important role in obesity is physical inactivity [9]. Previous studies have revealed that obese individuals had reduced physical activity [10] and a half of obese individuals had low cardiorespiratory fitness [11]. In addition, obese people with low cardiorespiratory fitness are at high risk of cardiovascular disease [12]. Taken together, reducing fat accumulation and increasing physical activity are likely to promote health benefits and minimize the risk of cardiovascular disease in this population.

The American College of Sport Medicine has recommended that aerobic exercise (AER) with moderate intensity for at least 150 min a week (30 min/day, 5 days/week) is optimal for individuals with obesity [13] due to body fat reduction [14,15,16] and cardiorespiratory fitness improvement [15,16]. Despite various health benefits, active exercise may be problematic in some obese people due to high body-mass index, low perceived motivation [17,18,19], and low cardiorespiratory fitness [12]. Thus, people with obesity with these barriers may have low adherence to exercise and be unable to achieve the targeted health benefits of exercise.

Whole-body vibration (WBV), functioning as a passive exercise, is an alternative therapeutic exercise. Vibration is a mechanical stimulus characterized by oscillatory motion, generating force to the muscle. Several systematic reviews and review articles have documented the effect of WBV in individuals who have musculoskeletal and neuromuscular deficits. Of these, WBV has the potential to gain bone mineral density in women with postmenopausal osteoporosis [20], improve neuromuscular performance [21], and enhance motor function in neurological disorders [22]. While this literature points out several health advantages, little is known about the effects of WBV on metabolic-related outcomes. Most previous studies conducted in animal models have evident beneficial effects of WBV on lipid mobilization, such as lowering cholesterol and triglycerides [23,24,25]. In addition, a previous study focusing on the acute effects of warm-up with vibration before (AER) on fat oxidation found that an additional WBV during warm-up had greater fat oxidation than warm-up without vibration in obese men [26]. However, no study has compared the effects of AER with WBV on metabolic-related outcomes in obesity. Regarding low cardiorespiratory fitness in obesity, a recent study suggested a vibration frequency at 30 Hz for obesity due to a non-impact effect on cardiovascular parameters [27]. It is likely that WBV at this frequency would be an advantage to obesity, especially in individuals who are unfit. Thus, this study aims to investigate the effects of AER and WBV on fat oxidation, lipid mobilization, cardiovascular responses, and dyspnea level in obesity.

## 2. Materials and Methods

### 2.1. Study Design and Participants

The study employed a randomized crossover design and aimed to recruit individuals who were dealing with obesity as its primary participant pool. In order to be eligible for participation, individuals were required to meet specific inclusion criteria, which included: (i) having a body-mass index (BMI) equal to or greater than 25 kg per square meter (kg/m^2^), as per the guidelines established for the Asia-Pacific region [28]; (ii) falling within the age range of 20 to 60 years; and (iii) demonstrating the capability to ambulate independently, ensuring self-sufficiency in terms of mobility. Any participants with uncontrolled cardiovascular diseases (such as unstable angina and acute heart failure), neurological disorders (such as severe spasticity and epilepsy), musculoskeletal conditions (such as severe osteoarthritis and rheumatoid arthritis), or respiratory diseases that significantly restrict their ability to perform aerobic exercise (AER) or engage in whole-body vibration (WBV, AMAXS AEX 2022, Chase Co., Ltd., Bangkok, Thailand), were expressly excluded from the study. Additionally, individuals who declined to participate in the research were not considered to be participants. Before commencing the study, all participants were required to provide their informed consent, signifying their willingness to engage in the research process while fully understanding its implications and potential risks. It is essential to note that the study was conducted under the ethical oversight and approval of the Research Ethics Committee at the Research Institute for Health Sciences, Chiang Mai University, with the official approval number denoted as 55/2022.

The sample size for this study was calculated, primarily based on a key primary outcome, fat oxidation, which was informed by a comprehensive review of a prior study [26]. In order to determine the required sample size, the mean and standard deviation of fat oxidation values were considered for both the group subjected to whole-body vibration (WBV, AMAXS AEX 2022, Chase Co., Ltd., Bangkok, Thailand) and the group without WBV. Specifically, the fat oxidation values were found to be 0.57 ± 0.12 g/min for the WBV group and 0.45 ± 0.12 g/min for the non-WBV group. These data were instrumental in gauging the between-group differences.

With 80% power, 5% type I error, and a large effect size of 0.5, a minimum sample size of 26 individuals was required to detect a statistically significant difference between the groups. In an abundance of caution and to ensure that the study had adequate statistical power to identify meaningful differences across various outcomes, the effect size was adjusted to the lower end of the range, specifically 0.25. This adjustment aimed to account for a potentially wider spectrum of between-group differences and their effects on the study variables. Consequently, a total of 98 participants were recruited into the study, representing a sufficiently robust and diverse sample size to yield meaningful results.

### 2.2. Intervention Protocols

Individuals meeting the eligibility criteria were randomly assigned to undertake two distinct exercise regimens: aerobic exercise (AER) and whole-body vibration (WBV, AMAXS AEX 2022, Chase Co., Ltd., Bangkok, Thailand). This random allocation was administered through a computerized generation process, ensuring an unbiased distribution of participants in the two exercise groups. To mitigate the potential of a “carry-over effect” from one exercise regimen to the other, a well-considered approach was adopted. A 1-week washout period was implemented between the completion of one exercise regimen and the commencement of the next. This interval provided a sufficient duration for any residual effects or influences from the previous regimen to dissipate.

To maintain consistency and minimize potential confounding factors from the laboratory environment, the laboratory room was rigorously maintained at a relative humidity level ranging from 50% to 55%, creating an environment conducive to participant comfort and wellbeing. Simultaneously, a constant room temperature of 25 °C was upheld, ensuring a stable and standardized climate throughout the study.

#### 2.2.1. Aerobic Exercise (AER)

Aerobic exercise (AER) was performed using a cycle ergometer (Lode, Lode B.V., Groningen, The Netherlands). AER included a warm-up session, the aerobic exercise session, and a cool-down session. Participants performed the warm-up by 5-min stretching of the hip flexor muscle group, knee extensor muscle group, and knee flexor muscle group (15 × 3 s/side/muscle group, both sides), before starting AER. For the exercise session, participants were asked to perform moderate intensity exercise at a constant speed of 60 revolutions per minute (rpm) for 30 min. Moderate intensity was determined to be 64–76% predicted maximum heart rate [29]. During an initial 5-min exercise, the workload was adjusted incrementally at every minute until moderate intensity was achieved. The workload (watt) was determined using Wasserman’s formula as follows: (VO2peak ((height − age) × sex) − VO2unloaded (150 + (6 × weight)))/100 [30]. The cool-down session was performed as the warm-up session described.

#### 2.2.2. Whole-Body Vibration Exercise (WBV)

The whole-body vibration (WBV) protocol employed in this study consisted of a structured sequence comprising three different sessions: a warm-up session, the vibration exercise session, and a cool-down session. The warm-up and cool-down sessions were carried out by stretching (same as mentioned in the AER protocol). Subsequently, participants were given precise instructions to assume a barefoot stance on the specialized vibration plate, specifically the AMAXS AEX 2022 model manufactured by Chase Co., Ltd., located in Bangkok, Thailand. In this position, participants were instructed to stand with their feet positioned apart, maintaining a width equal to the span between their shoulders, and their knees flexed at an angle ranging from 150 to 170 degrees. Importantly, a stabilized walker aid was placed in front of participants, serving as a safety precaution to prevent potential falls. Although this safety measure was in place, none of the participants found it necessary to use the walker aid during the actual exercise session. Within the context of the vibration exercise itself, all participants were asked to perform the exercises at a consistent frequency of 30 Hz. The exercise regimen was structured with a 1-min bout of vibration exercise, followed by a 1-min period of rest, and this pattern alternated throughout the 30-min duration of the exercise session.

### 2.3. Outcome Measures

#### 2.3.1. Measurement of Fat Oxidation

The assessment of participants’ metabolic activity and energy expenditure during the study was conducted through the application of indirect calorimetry. This technique, carried out using a stationary metabolic cart (the Corival CPET system, manufactured by Lode B.V. in Groningen, The Netherlands), enabled the measurement of concentrations of oxygen consumption and carbon dioxide production.

By analyzing fat oxidation, the concentrations of both oxygen and carbon dioxide gases were used to calculate the rate of fat oxidation utilizing the following equation: fat oxidation rate (expressed in grams per minute, g/min) = the volume of oxygen consumed (VO2) multiplied by 1.695, minus the volume of carbon dioxide produced (VCO2), multiplied by 1.701 [31]. In addition to fat oxidation, the cumulative fat oxidation rate was determined to gain a comprehensive understanding of fat utilization over time. This cumulative measure encapsulated total fat oxidation from the baseline assessment through to the end of each exercise regimen.

#### 2.3.2. Measurement of Lipid Mobilization

To assess lipid mobilization, the research study focused on triglyceride (TG) levels in participants. Blood samples from each participant were taken in the morning, ensuring consistency in the timing of sample collection. These blood samples, totaling 10 milliliters in volume, were drawn by a qualified medical technologist, guaranteeing the precision and integrity of the samples. The collected blood samples were preserved in specialized tubes containing a clot activator and sodium fluoride (NaF). Following collection, these samples underwent a controlled centrifugation process, which subjected them to a rotational speed of 2500 revolutions per minute (rpm) for a duration of 10 min. This centrifugation step facilitated the separation of the blood into two distinct components: serum and NaF plasma. These separated components were then kept at a temperature of −80 °C to ensure their preservation and maintain the stability of the lipid samples until the time of analysis. Subsequent to this sample processing, the concentration of TG was quantified using an enzymatic colorimetric method. This analysis was performed with the aid of an advanced and automated analyzer, specifically the apparatus manufactured by Biosystems S.A. in Barcelona, Spain.

#### 2.3.3. Measurements of Cardiovascular Parameters and Dyspnea Level

Cardiovascular parameters in this study included heart rate (HR) and blood pressure (systolic blood pressure (SBP) and diastolic blood pressure (DBP)). HR was monitored through the utilization of Polar technology, attached on the middle of each participant’s chest wall, ensuring real-time and accurate data collection. This equipment was connected to the CPET system (Corival CPET by Lode B.V., Groningen, The Netherlands), and facilitated the continuous monitoring and recording of heart rate data during the exercise regimens. Additionally, SBP and DBP were methodically assessed using blood pressure cuffs placed on each participant’s arm. The cuffs were also integrated into the CPET system and monitored throughout the exercise. This integration enabled the real-time measurement and recording of participants’ blood pressure data throughout the exercise sessions.

Furthermore, the dyspnea level, expressed by rating perceived exertion (RPE), was measured using the modified Borg scale (mBorg). This method is a valid and reliable tool for quantifying perceived effort and discomfort [32]. Participants were asked to rate their level of dyspnea on a scale ranging from 0 to 10; fewer scores represented a lower dyspnea level while higher scores indicated a higher dyspnea level.

### 2.4. Procedure

After completing the initial screening process, a total of 49 eligible participants were enrolled in this study, each participating in two separate visits. During the first visit, participants were asked for their demographic data. These data included age, gender, weight, height, body-mass index (BMI), waist circumference, hip circumference, underlying medical conditions, and an evaluation of their physical activity level. The assessment of physical activity level was carried out using a short version of the International Physical Activity Questionnaire (IPAQ) [33]. To establish a consistent baseline, participants were asked to sit quietly in a controlled laboratory room, with a 5-min rest period before the commencement of the exercise regimen. This period of rest was instrumental in ensuring that participants began the exercise protocol in a state of rest, minimizing any confounding factors. Following the period of rest, participants were instructed to perform the randomized exercise protocol, lasting for a duration of 30 min. Blood samples were collected both before the commencement and immediately following the conclusion of the exercise session. Measurements of VO2, VCO2, all cardiovascular parameters (i.e., heart rate and blood pressure), and the level of perceived dyspnea were recorded at 5-min intervals throughout the exercise session.

**Figure 1:**
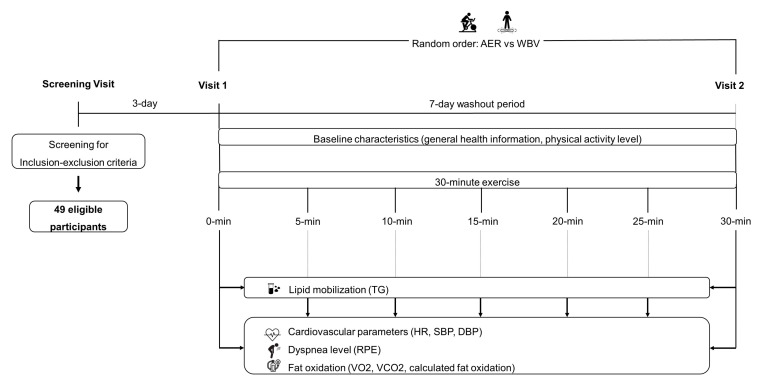
The study procedure. AER, aerobic exercise; DBP, diastolic blood pressure; HR, heart rate; min, minute; RPE, rating perceived exertion; SBP, systolic blood pressure; TG, triglycerides; VCO2, carbon dioxide production; VO2, oxygen consumption; WBV, whole-body vibration.

For the second visit, all participants were instructed to perform the remaining exercise protocol, mirroring the procedure in the first visit. To ensure the consistency and reliability of the data collected, participants were asked to adhere to a set of stringent pre-experiment conditions, refraining from engaging in strenuous exercise for a minimum of 24 h, avoiding the consumption of alcohol and smoking for a similar duration, and fasting for a minimum of 12 h prior to the experiment day. The study procedure is shown in Figure 1.

### 2.5. Statistical Analyses

All data were analyzed using Statistical Package for Social Sciences (SPSS), version 17.0 for Windows. Descriptive statistics were used to present demographic data. Data distribution was assessed using the Shapiro-Wilk test. A two-way mixed analysis of variance (ANOVA) was used to determine the differences between and within groups in TG levels, fat oxidation rate, cardiovascular parameters, and dyspnea level, with Bonferroni post-hoc adjustment for the subgroup analysis. Between-group differences in cumulative fat oxidation were computed using a dependent sample *t*-test. Subgroup analyses by age and sex were also taken. Based on participants in this study, three subgroups (i.e., males aged 20–45, females aged 20–45, and females aged 45–60) were analyzed. All outcomes were expressed as mean ± standard deviation. The significance level was less than 0.05.

## 3. Results

Of 49 eligible participants, one participant was removed from the study due to being unable to complete the aerobic exercise trial. Thus, a total number of 48 participants were analyzed. Most participants were female (72.9%), with mean age of 28.94 years. The three most underlying diseases were allergic diseases, treated dyslipidemia, and treated hypothyroidism, respectively. Most obese participants had a physical activity level of minimal active level activity. All demographic data are shown in Table 1.

### 3.1. Fat Oxidation and Lipid Mobilization

**Figure 2:**
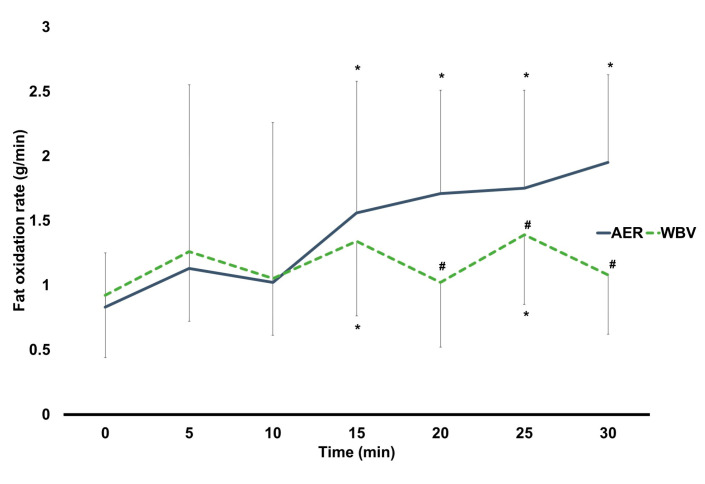
Changes within and between AER and WBV groups on fat oxidation. AER, aerobic. exercise; g/min, gram per minute; min, minute; WBV, whole-body vibration; #, between-group significant difference; *, significant difference when compared to baseline.

The results found a significant interaction effect of time × group on fat oxidation rate (F5.08, 477.47 = 9.04, *p* < 0.001). The post-hoc analysis found no between-group difference in fat oxidation rate at any time point, except for 20 min of exercise onwards, shown as significantly higher fat oxidation rates in AER than in WBV (AMAXS AEX 2022, Chase Co., Ltd., Bangkok, Thailand) (Figure 2). Regarding within-group changes, fat oxidation rate in the AER group was significantly higher at 15 min of exercise onwards compared to their baseline, while fat oxidation rate in the WBV (AMAXS AEX 2022, Chase Co., Ltd., Bangkok, Thailand) group was significantly greater at 15 and 25 min of exercise (Figure 2). The study further showed a significantly higher cumulative fat oxidation rate in the AER group than in the WBV (AMAXS AEX 2022, Chase Co., Ltd., Bangkok, Thailand) group (AER vs. WBV; 9.96 ± 4.30 vs. 8.06 ± 2.16 g/min; *p* = 0.002).

A subgroup of males aged 20–45 found significantly higher fat oxidation in AER than in WBV (AMAXS AEX 2022, Chase Co., Ltd., Bangkok, Thailand) at 20 and 30 min (*p* < 0.05 for all). Within-group analyses found that, compared to their baseline, there were significantly greater fat oxidation rates in AER from 20 min onwards while no changes in fat oxidation throughout the 30-min WBV (AMAXS AEX 2022, Chase Co., Ltd., Bangkok, Thailand). A subgroup of females aged 20–45 demonstrated significantly higher fat oxidation rates in AER than in WBV (AMAXS AEX 2022, Chase Co., Ltd., Bangkok, Thailand) from 20 min onwards (*p* < 0.05 for all). Within-group analyses found that AER had significantly higher fat oxidation rates from 20 min onwards compared to their baseline, while WBV (AMAXS AEX 2022, Chase Co., Ltd., Bangkok, Thailand) had significantly higher fat oxidation rates at 15 and 25 min (*p* < 0.05 for all). For a subgroup of females aged 45–60, fat oxidation rates were significantly higher in AER than in WBV (AMAXS AEX 2022, Chase Co., Ltd., Bangkok, Thailand) at 20 and 30 min of exercise (*p* < 0.05 for all). AER had significantly higher fat oxidation rates at 5, 25, and 30 min compared to their baseline (*p* < 0.05 for all), whereas no changes in fat oxidation at any time points during WBV (AMAXS AEX 2022, Chase Co., Ltd., Bangkok, Thailand) were observed (Table 2).

**Figure 3:**
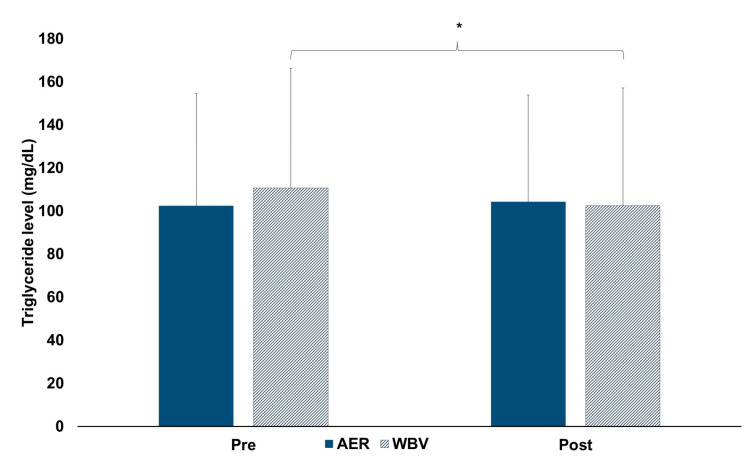
Changes within and between AER and WBV groups on triglyceride levels. AER, aerobic exercise; mg/dL, milligram per deciliter; Pre, pre-test; Post, post-test; WBV, whole-body vibration; *, significant pre-post difference.

For lipid mobilization, a significant interaction effect of time x group on TG levels (F1, 94 = 8.59, *p* = 0.004) was reported. After the post-hoc analysis, there was no difference in TG levels between groups (Figure 3). Regarding within-group changes, TG levels were 7.4% significantly lower after completing 30 min of WBV (AMAXS AEX 2022, Chase Co., Ltd., Bangkok, Thailand) (pre vs. post: 110.71 ± 55.56 vs. 102.5 ± 54.62, *p* = 0.001), while there was no change in TG levels after 30 min of AER (pre vs. post: 102.25 ± 52.30 vs. 104.15 ± 49.70) (Figure 3).

For a subgroup of males aged 20–45, there was no difference between AER and WBV (AMAXS AEX 2022, Chase Co., Ltd., Bangkok, Thailand). TG levels were only significantly lower after completing 30 min of WBV (*p* = 0.012). Regarding a subgroup of females aged 20–45, the results showed that there was no between-group difference in TG levels. TG levels were significantly lower after completing 30 min of WBV (AMAXS AEX 2022, Chase Co., Ltd., Bangkok, Thailand) (*p* = 0.045), but not in AER. For a subgroup of females aged 45–60, there were no between and within AER and WBV (AMAXS AEX 2022, Chase Co., Ltd., Bangkok, Thailand) group differences in TG levels. (Table 2).

### 3.2. Cardiovascular Parameters and Dyspnea Level

**Figure 4:**
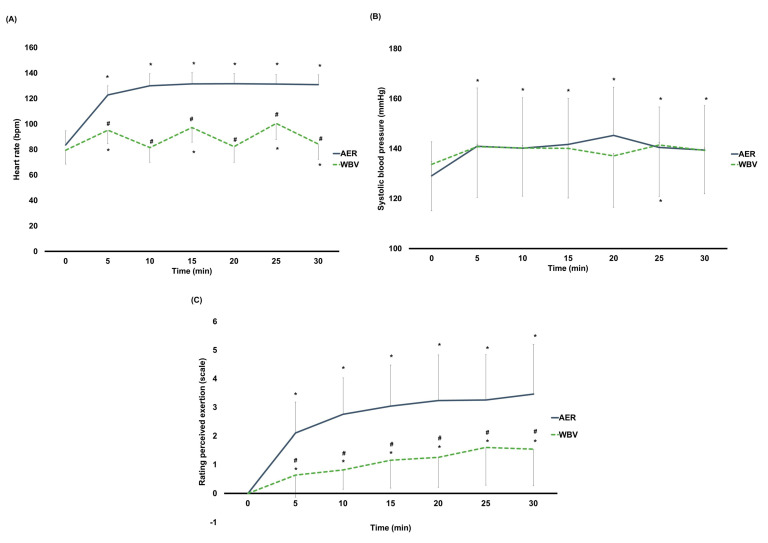
(**A**) Changes within and between AER and WBV groups on heart rate. (**B**) Changes within and between AER and WBV groups on systolic blood pressure. (**C**) Changes within and between AER and WBV groups on rating perceived exertion. AER, aerobic exercise; bpm, beat per minute; min, minute; mmHg, millimeter of mercury; WBV, whole−body vibration; #, between−group significant difference; *, significant difference when compared to baseline.

Significant interaction effects (time × group) on HR (F3.48, 327.11 = 219.03, *p* < 0.001), SBP (F6, 564 = 2.33, *p* = 0.03), and RPE (F2.45, 229.97 = 21.55, *p* < 0.001) were observed. Except for baseline, HR was significantly higher after AER than WBV (AMAXS AEX 2022, Chase Co., Ltd., Bangkok, Thailand) at all time points (*p* < 0.001 for all) (Figure 4A) (mean HR between AER vs. WBV (AMAXS AEX 2022, Chase Co., Ltd., Bangkok, Thailand) at the end of exercise: 130 ± 8 vs. 84 ± 12 bpm). Within-group differences found that HR in the AER group was significantly increased throughout the exercise (*p* < 0.001 for all), while HR in the WBV (AMAXS AEX 2022, Chase Co., Ltd., Bangkok, Thailand) group was only significantly greater at 5, 15, 25, and 30 min of vibration exercise (Figure 4A). No differences in SBP between groups were found (Figure 4B). SBP in the AER group was significantly raised throughout the exercise (*p* < 0.05 for all) (mean SBP between AER vs. WBV (AMAXS AEX 2022, Chase Co., Ltd., Bangkok, Thailand) at the end of exercise: 139 ± 18 vs. 139 ± 17 mmHg), while no changes in SBP in the WBV (AMAXS AEX 2022, Chase Co., Ltd., Bangkok, Thailand) group were observed (Figure 4B). In line with the HR, RPE was significantly higher in the AER group than in the WBV (AMAXS AEX 2022, Chase Co., Ltd., Bangkok, Thailand) group at all time points (*p* < 0.001 for all) (Figure 4C) (mean RPE between AER vs. WBV (AMAXS AEX 2022, Chase Co., Ltd., Bangkok, Thailand) at the end of exercise: 3.5 vs. 1.5). RPE in both groups was significantly higher compared to their baseline (*p* < 0.001 for all) (Figure 4C). Subgroup analyses by age and sex showed similar results of all cardiovascular parameters and dyspnea level to the main analyses.

## 4. Discussion

Fat accumulation and physical inactivity are risk factors for developing cardiovascular disease in individuals with obesity [4,5,12]. A previous study has suggested a higher cardiovascular disease-related mortality in obesity than in those with normal weight, and even the highest cardiovascular disease-related mortality in individuals with obesity who had low physical activity [34]. Aerobic exercise has been recommended as a suitable therapeutic approach for obesity to lower body fat [14,15,16] and improve cardiorespiratory fitness [15,16], consequently reducing cardiovascular risks in this population. Since aerobic exercise needs individual’s effort, some people with obesity may not benefit from this type of exercise due to several limitations (i.e., huge weight and poor cardiorespiratory endurance) to perform the aerobic exercise [12,17,18,19]. Thus, other therapeutic strategies are required. Whole-body vibration therapy is one alternative exercise, functioning as a passive exercise. It would be better if whole-body vibration promoted similar health benefits to aerobic exercise. To our knowledge, this is the first study comparing the effects of aerobic exercise and whole-body vibration on metabolism and cardiovascular responses in individuals with obesity.

Findings of this study reveal a greater fat oxidation in AER than in WBV (AMAXS AEX 2022, Chase Co., Ltd., Bangkok, Thailand), with increased fat oxidation in both AER and WBV (AMAXS AEX 2022, Chase Co., Ltd., Bangkok, Thailand). Females aged below 45 but not males had similar responses to such findings. Unlike fat oxidation, WBV (AMAXS AEX 2022, Chase Co., Ltd., Bangkok, Thailand) had a similar effect on lipid mobilization to AER, with an increased lipid mobilization after WBV (AMAXS AEX 2022, Chase Co., Ltd., Bangkok, Thailand). Regardless of sex, those aged below 45 had the same responses to the main findings. Regarding cardiovascular responses and dyspnea level, similar responses on systolic blood pressure and less impact on heart rate activity and dyspnea level were observed in WBV (AMAXS AEX 2022, Chase Co., Ltd., Bangkok, Thailand) than in AER.

During moderate intensity of aerobic exercise, fatty acids are produced via lipolysis to meet the energy demand [35]. In our study, the superior effect on fat oxidation in AER indicates its capability to stimulate more fat utilization than in WBV (AMAXS AEX 2022, Chase Co., Ltd., Bangkok, Thailand). AER induces a release of fatty acids into the blood circulation in response to the increased fuel demand. In addition, a greater cumulative fat oxidation in AER confirms better stimulation than in WBV (AMAXS AEX 2022, Chase Co., Ltd., Bangkok, Thailand). The better response to fat oxidation in AER is attributed to the different type of exercise. AER is characterized as a continuous exercise, while WBV (AMAXS AEX 2022, Chase Co., Ltd., Bangkok, Thailand) is performed as an alternating exercise. This study notes that fat was more oxidized during vibrating period than the rest interval, thereby resulting in less cumulative fat oxidation in WBV (AMAXS AEX 2022, Chase Co., Ltd., Bangkok, Thailand) than in AER. Despite the inferior effect of WBV (AMAXS AEX 2022, Chase Co., Ltd., Bangkok, Thailand) on fat oxidation compared to AER, WBV (AMAXS AEX 2022, Chase Co., Ltd., Bangkok, Thailand) also promotes fat oxidation, as reflected by an increase in fat oxidation during WBV (AMAXS AEX 2022, Chase Co., Ltd., Bangkok, Thailand). This finding is supported by previous work demonstrating that warm-up with whole-body vibration gained more fat oxidation compared to no vibration in obese men [26]. Our study further illustrates that females aged below 45 exhibited similar fat oxidation responses to the main finding. In contrast, those aged 45 and above showed unchanged fat oxidation during WBV (AMAXS AEX 2022, Chase Co., Ltd., Bangkok, Thailand). Indeed, fat oxidation decreases with advancing age during exercise, partly due to lower fat-free mass [36]. Increased fat mass and decreased fat-free mass contribute to impaired substrate oxidative capacity via a reduction in mitochondrial fatty acid uptake [37]. It is possible that WBV (AMAXS AEX 2022, Chase Co., Ltd., Bangkok, Thailand) may limit the activation of fat oxidation in obese individuals with increasing age. This limitation in fat oxidation may also be attributable to impaired estrogen function [36], given that all participants in this group were menopausal females. Sex-based differences are also observed after eliminating age-related effects on fat oxidation. Our study underscores a greater fat oxidation throughout a WBV (AMAXS AEX 2022, Chase Co., Ltd., Bangkok, Thailand) session in females aged below 45, but not in males. This finding is supported by the previous literature describing a greater percentage of intramyocellular lipids in females than in males [38]. This suggests that females are likely to have a larger reliance on fat sources than males, as a consequence of their greater capacity to utilize intramyocellular lipids.

Similar effects on lipid mobilization, as reflected by a non-significant difference in triglyceride levels between post-AER and post-WBV (AMAXS AEX 2022, Chase Co., Ltd., Bangkok, Thailand), suggests an equal response. Interestingly, triglyceride levels were lower after completing a WBV (AMAXS AEX 2022, Chase Co., Ltd., Bangkok, Thailand) session. This finding is in agreement with previous studies showing a reduction in triglyceride levels after WBV training in obese and old mice [23,24,25]. A vibration stimulus generates force to the muscle via oscillatory motion, leading to muscle activation [39]. Reduction in triglyceride levels after completing WBV (AMAXS AEX 2022, Chase Co., Ltd., Bangkok, Thailand) probably results from the enhanced energy demand, which aggravates triglyceride utilization as a substrate. While triglyceride levels were lower after WBV (AMAXS AEX 2022, Chase Co., Ltd., Bangkok, Thailand), it is doubted why triglyceride levels after AER were unchanged. Possibly, baseline values in the AER group were lower than those in the WBV group (but no significant difference between groups), leading to little room for change within the AER group.

Poor cardiorespiratory fitness in individuals with obesity is one of the factors contributing to not engaging in aerobic exercise [12]. WBV, represented as a passive exercise with a frequency at 30 Hz, has suggested a slight interference on cardiovascular parameters in obesity [27]. Our study confirms a low impact on cardiovascular parameters, as indicated by a lower heart rate during WBV (AMAXS AEX 2022, Chase Co., Ltd., Bangkok, Thailand) than during AER and non-significant changes in systolic blood pressure throughout WBV (AMAXS AEX 2022, Chase Co., Ltd., Bangkok, Thailand). Similar to heart rate response, our study also shows a lower dyspnea level in WBV (AMAXS AEX 2022, Chase Co., Ltd., Bangkok, Thailand) than in AER; a lower dyspnea level in WBV (AMAXS AEX 2022, Chase Co., Ltd., Bangkok, Thailand) than in AER, as reflected by the very light to light dyspnea level at the end of WBV (AMAXS AEX 2022, Chase Co., Ltd., Bangkok, Thailand) (RPE = 1.5) and moderate to little intense dyspnea level at the end of AER (RPE = 3.5), was observed. A consistent result between heart rate and dyspnea responses is supported by previous evidence establishing a strong correlation between these two parameters during incremental exercise [40]. Thus, the low impact on heart rate and less psychological strain, as indicated by RPE [40], is likely to enhance exercise adherence, thereby reaching exercise benefits during WBV (AMAXS AEX 2022, Chase Co., Ltd., Bangkok, Thailand).

Some limitations of this study should be noted. First, metabolic and cardiovascular changes are acutely responded to a single bout. Further study needs to confirm the physiological adaptation in response to WBV training at a specific frequency of 30 Hz. Second, the majority of participants in our study were females and young adults, which may limit generalizability. Lastly, other lipid profiles, such as low density-lipoprotein (LDL) and cholesterol, which are also accounted as essential cardiovascular risk factors in obesity, were not measured. We cannot speculate whether other lipid profiles and triglycerides have similar responses to WBV.

## 5. Conclusions

In summary, WBV (AMAXS AEX 2022, Chase Co., Ltd., Bangkok, Thailand) at a frequency of 30 Hz had an inferior effect on fat oxidation, an equal effect on lipid mobilization, and a superior effect on cardiovascular responses compared to AER in individuals with obesity. The results further showed that WBV (AMAXS AEX 2022, Chase Co., Ltd., Bangkok, Thailand) was able to induce fat oxidation (particularly in females aged below 45) and lipid mobilization, and reduce interference on cardiovascular parameters during WBV (AMAXS AEX 2022, Chase Co., Ltd., Bangkok, Thailand). Thus, WBV (AMAXS AEX 2022, Chase Co., Ltd., Bangkok, Thailand) may reduce fat accumulation and facilitate lipid mobilization in obesity. WBV (AMAXS AEX 2022, Chase Co., Ltd., Bangkok, Thailand) may be employed as an alternative exercise instead of aerobic exercise.

## Figures and Tables

**Table 1:** Demographic data.

Variables		N = 48 (Mean ± SD)
Age (year)		28.94 ± 11.39
Sex (males, %)		27.1
Weight (kg)		80.52 ± 19.31
Height (cm)		163.10 ± 9.88
Body-mass index		30.03 ± 5.58
Waist circumference (cm)		91.73 ± 14.99
Hip circumference (cm)		111.19 ± 15.17
Underlying diseases (*n*, %)	Allergic diseases	6 (12.50)
	Treated dyslipidemia	3 (6.25)
	Treated hypothyroidism	2 (4.17)
	Others	5 (10.42)
Physical activity level (*n*, %)	Inactive level	16 (33.33)
	Minimal active level	23 (47.92)
	High active level	9 (18.75)

**Table 2:** Fat oxidation and triglyceride levels between AER and WBV by age and sex.

Variables	Time (min)	AER	WBV
Fat oxidation (g/min)			
Males aged 20–45 (*n* = 13)	0	0.77 ± 0.46	1.10 ± 0.60
	5	0.56 ± 1.52	1.21 ± 0.78
	10	1.23 ± 1.89	1.04 ± 0.60
	15	1.56 ± 1.38	1.34 ± 0.92
	20	1.90 ± 0.80 *	0.97 ± 0.71 #
	25	1.93 ± 0.96 *	1.49 ± 0.74
	30	2.28 ± 0.75 *	0.96 ± 0.59 #
Females aged 20–45 (*n* = 30)	0	0.89 ± 0.42	0.83 ± 0.42
	5	1.35 ± 1.46	1.27 ± 0.46
	10	1.01 ± 0.94	1.07 ± 0.34
	15	1.59 ± 0.92 *	1.35 ± 0.41 *
	20	1.68 ± 0.83 *	1.07 ± 0.39 #
	25	1.69 ± 0.71 *	1.36 ± 0.48 *#
	30	1.85 ± 0.64 *	1.15 ± 0.40 #
Females aged 45–60 (*n* = 5)	0	0.59 ± 0.28	0.95 ± 0.32
	5	1.45 ± 0.37 *	1.23 ± 0.31
	10	0.67 ± 0.65	0.64 ± 0.22
	15	1.19 ± 0.78	1.12 ± 0.42
	20	1.37 ± 0.55	0.71 ± 0.29 #
	25	1.54 ± 0.39 *	1.24 ± 0.30
	30	1.66 ± 0.39 *	0.92 ± 0.39 #
Triglyceride level (mg/dL)			
Males aged 20–45 (*n* = 13)	pre	126.38 ± 70.77	143.23 ± 72.15
	post	131.46 ± 64.05	129.77 ± 66.35 *
Females aged 20–45 (*n* = 30)	pre	89.17 ± 40.36	95.50 ± 40.96
	post	90.33 ± 38.87	88.97 ± 45.64 *
Females aged 45–60 (*n* = 5)	pre	118.0 ± 42.69	117.4 ± 57.18
	post	116.0 ± 41.62	112.80 ± 53.10

AER, aerobic exercise; g/min, gram per minute; min, minute; mg/dL, milligram per deciliter; Pre, pre-test; Post, post-test; WBV, whole-body vibration; #, between-group significant difference; *, significant difference when compared to baseline.

## Data Availability

The data presented in this study are available upon request from the corresponding authors.
